# 

**Published:** 1913-08

**Authors:** 


### The Woman's Department

**CONDUCTED BY MRS. F. E. DANIEL**

COLLABORATORS

Dr. J. S. Abbott, Dairy and Food Commissioner of Texas; Miss Eleanor Brackenridge, President of the Equal Franchise Society, San Antonio; Dr. G. Henri Bogart, Associate Editor, Utah Medical Journal, Paris, Ill.; Mrs. Isabella Charles Davis, Second Vice President National Order of "King's Daughters," New Jersey; Mrs. Jno. S. Turner, Recording Secretary, Texas Congress of Mothers; Dr. Marvin B. Grace, Seguin; Dr. Margaret Holliday, Women's Physician, University of Texas, Austin; Dr. John S. Turner, President State Medical Association; Dr. Ralph Steiner, State Health Officer; Dr. L. H. Sackett, Instructor in the Philosophy of Education, University of Texas; Dr. F. S. White, Supt. S. W. Texas Lunatic Asylum, San Antonio, Texas; Mrs. M. C. Kersh, Dallas, Texas.

### VITAL FACTS EVERY WOMAN SHOULD KNOW.

*Teach me the way to a strong body, a pure mind, a noble character.*

*"Ignorance is not innocence."*

